# Mapping the Research Landscape of Exercise and Breast Cancer: A Bibliometric Analysis From 2020 to 2024

**DOI:** 10.1155/ijog/2972077

**Published:** 2025-10-23

**Authors:** Jian Li, LiYing Qiang, MingYue Jiao, BinBin Zhang, Pengpeng Zhang

**Affiliations:** ^1^ Department of Physical Education, Shanxi Institute of Science and Technology, Jincheng, Shanxi, China; ^2^ Faculty of Education and Liberal Arts, INTI International University, Nilai, Negeri Sembilan, Malaysia, newinti.edu.my; ^3^ Department of Physical Education, Shanxi University of Electronic Science and Technology, Linfen, Shanxi, China; ^4^ School of Teacher Education, Hezhou University, Hezhou, Guangxi, China, hzu.gx.cn; ^5^ Faculty of Sports and Leisure, Guangdong Ocean University, Zhanjiang, Guangdong, China, gdou.edu.cn

**Keywords:** bibliometric analysis, breast cancer, exercise oncology, immune modulation, survivorship interventions

## Abstract

Even though there is strong evidence that exercise improves breast cancer patients′ quality of life, treatment tolerance, and survival, disparate research initiatives and uneven clinical acceptance underscore the need for a thorough synthesis of trends, partnerships, and gaps. This bibliometric analysis systematically maps the research landscape of exercise and cardiovascular disease (CVD) from 2020 to 2024, aiming to identify trends, influential works, and knowledge gaps in this critical field. Utilizing data from the Web of Science (WoS) and VOSviewer, the study analyzed 314 articles through cocitation, coword, and co‐occurrence analyses. Key findings reveal exponential growth in citations, driven by seminal guidelines (e.g., ESC 2021 and WHO 2020) and studies on sedentary behavior and pandemic‐related activity changes. The intellectual structure is anchored by three themes: evidence‐based guidelines, risk quantification (e.g., dose–response relationships), and behavioral interventions. Dominant journals include the *British Journal of Sports Medicine* and *JAMA*, while influential authors like Dominique Hansen and Emmanuel Stamatakis shape the field. Geographic disparities highlight Western‐centric research dominance, though emerging contributions from Asia and Europe are noted. The study identifies gaps in mechanistic research, personalized exercise prescriptions, and equitable translation of evidence into practice. Practical implications call for standardized clinical protocols, dual public health messaging (activity promotion and sedentary reduction), and digital health integration. This analysis provides a roadmap for future research, emphasizing transdisciplinary collaboration to optimize CVD prevention through physical activity.

## 1. Introduction

Adjunctive medicines are becoming more and more important in the fight against breast cancer, which is still one of the most common cancers in the world [1]. With notable improvements in quality of life, treatment tolerance, and survival rates, physical activity has become a crucial nonpharmacological intervention in recent years [2, 3]. According to research, organized exercise programs, such as resistance and aerobic training, can lower inflammation [4], modify immunological function [5], and lessen treatment‐related adverse effects such as fatigue and lymphedema [6]. The increasing amount of data highlights the importance of exercise in the treatment of breast cancer, necessitating systematic reviews to chart the field′s changing research terrain.

The field is plagued by fragmented research efforts and poor clinical application, despite the documented benefits of exercise in the treatment of breast cancer [7, 8]. Important gaps include differences in accessibility and adherence among distinct populations [9] and disagreements over the best exercise prescriptions (e.g., kind, intensity, and duration) for various therapy phases [10]. Furthermore, whereas mechanistic research on microRNAs (e.g., [11]) reveals biological pathways, there is still little application of these findings in clinical settings. To combine these disparate findings, pinpoint research trends, and rank future studies to close the gap between theory and practice, a methodical bibliometric study is therefore crucial.

Both academic research and clinical oncology can benefit greatly from this bibliometric approach. It will identify the most influential research themes (e.g., exercise immunology and survival interventions) and collaboration networks by methodically analyzing publications from 2020 to 2024 [12, 13]. Funding allocation, policy formation, and the creation of precision‐based exercise programs suited to tumor subtypes and treatment methods can all be guided by these findings [14, 15]. In order to provide a more fair integration of exercise oncology into routine care, it will also draw attention to understudied areas, such as the role of exercise in metastatic disease or socioeconomically disadvantaged individuals.

There are several obstacles in the way of improving exercise oncology. Reproducibility and comparability are hampered by methodological diversity in study designs, such as differing definitions of exercise duration and intensity [16]. Logistical obstacles (such as cost and facility access) and a lack of clinician involvement in prescribing physical activity are the key reasons why long‐term adherence to exercise regimens is still poor. Furthermore, whereas bibliometric tools like VOSviewer make it possible to see trends, they could ignore contextual elements such as cultural variations in the norms surrounding physical activity or the uptake of telehealth during a pandemic [17]. To guarantee that exercise treatments reach a variety of global populations, addressing these issues calls for standardized reporting standards, multidisciplinary collaboration, and equitable implementation strategies [18].

## 2. Literature Review

### 2.1. Clinical Trials on Exercise Prescription

The best quality of evidence for developing exercise recommendations in the treatment of breast cancer comes from randomized controlled trials, or RCTs. A supervised program that combined aerobic and resistance exercise during adjuvant chemotherapy significantly improved cardiorespiratory fitness, muscular strength, and quality of life as compared to normal treatment, according to the seminal study by Courneya et al. [2]. Building on this, a dose–response RCT by An et al. [10] demonstrated that strenuous activity for 75–150 min/week resulted in better long‐term patient‐reported outcomes and health‐related fitness than recommended. The international consensus statement by Campbell et al. [7] emphasizes the necessity for customized prescriptions based on treatment phase, toxicity profiles, and specific patient characteristics, but it also highlights the difficulties that still exist in finding the best exercise parameters. Although further study is required to improve implementation techniques, these trials collectively highlight the importance of exercise as part of comprehensive cancer care.

### 2.2. Mechanistic and Immunomodulation Studies

The biochemical processes behind the antitumor effects of exercise have been clarified by recent translational research. In breast cancer models, aerobic exercise training changed the tumor microenvironment by increasing CD8+ T‐cell infiltration through CXCR3 signaling pathways and, as a result, improving responsiveness to immune checkpoint inhibitors, according to groundbreaking results presented by Gomes‐Santos et al. [5]. The clinical results of Hiensch et al. [4] showed that exercise‐mediated decreases in systemic inflammation, specifically IL‐6 and CRP levels, were associated with a notable improvement in fatigue in patients receiving treatment, which supported this immunomodulatory impact. Pleiotropic effects on tumor biology are suggested by other mechanistic investigations, such as the work of Falzone et al. [11] on exercise‐induced miRNA regulation. According to these findings, exercise can be used as a supplement to contemporary systemic medicines rather than only as supportive care, which calls for more research into how it works in tandem with immunotherapy regimens and targeted treatments.

### 2.3. Meta‐Analyses and Systematic Reviews

Extensive meta‐analyses have confirmed the therapeutic effects of exercise in the treatment of breast cancer. After doing a thorough analysis of data from 20 prospective trials, Spei et al. [19] showed that physically active survivors had a substantial 24% lower all‐cause mortality rate. In a similar vein, Hasenoehrl et al. [6] found that progressive resistance training decreased the incidence of lymphedema by 38% when compared to usual therapy after conducting a thorough meta‐analysis of 15 studies. Even while these extensive studies offer strong proof of the effectiveness of exercise, they frequently point out important knowledge gaps, especially with regard to metastatic patients who make up fewer than 10% of the cohorts under study [20]. These syntheses′ methodological rigor underscores the need for more inclusive research populations while fortifying the body of data in exercise oncology.

### 2.4. Guidelines and Implementation Research

For cancer survivors, current clinical guidelines, such as the groundbreaking ACSM roundtable [8], consistently urge ≥ 150 min of moderate exercise per week. Implementation studies, however, show significant real‐world obstacles. For example, Cormie et al. [21] discovered that in community settings, only 35% of patients achieved these goals, highlighting the significance of accessible programs. According to Patel et al. [15], patients with low incomes had 2.5 times more difficulty accessing exercise resources. Hospital–community partnerships and digital platforms are examples of innovative solutions that are emerging, but fundamental policy reforms are still necessary to enable equitable implementation. These results highlight how critical it is to close the gap between clinical practice and evidence‐based recommendations, especially for marginalized groups.

### 2.5. Understudied Populations and Future Directions

The majority of current exercise oncology research focuses on patients with early‐stage breast cancer, leaving important gaps in knowledge about its effects for elderly populations (≥ 65 years represent < 15% of cohorts) and metastatic patients (< 5% of trial participants) [22]. Novel intervention techniques are highlighted by emerging research, such as the randomized experiment conducted by Khodabakhshi et al. [23], which showed that combining ketogenic diets with exercise routines improved quality‐of‐life results. Furthermore, van Waart et al. [24] reported 72% adherence rates using remote monitoring throughout chemotherapy, demonstrating the promise of technology‐mediated solutions. These marginalized groups should be given priority in future research as it examines (1) exercise–immune interactions in advanced disease, (2) culturally appropriate therapies for a range of socioeconomic groups, and (3) the use of wearable technology to customize prescriptions. These approaches have the potential to change the paradigms of supportive care for all patients with breast cancer.

## 3. Methodology

Recent analyses in comparable disciplines have shown that the bibliometric approach used in this work is especially well suited for mapping the quickly changing research landscape of exercise and breast cancer [12, 13]. This approach offers a macrolevel knowledge of the evolution of exercise oncology from 2020 to 2024—a time of notable advancements in precision medicine and survivorship care—by methodically assessing publishing trends, collaboration networks, and topic clusters. The trustworthiness of the findings is ensured by the inclusion criteria, which emphasize peer‐reviewed studies and remove nonscientific outputs. These criteria are consistent with stringent bibliometric techniques observed in previous analyses of cancer rehabilitation [25, 26]. In order to uncover gaps that directly impact future clinical and investigational objectives in breast cancer exercise therapies, such as the underrepresentation of resistance training studies or geographic discrepancies in research output, methodological rigor is crucial.

Finding hidden patterns in the literature requires analyzing cocitation and keyword co‐occurrence networks using sophisticated visualization tools (like VOSviewer). As demonstrated by bibliometric research on cancer‐related physical activity [17] and sports medicine [27, 28], these methods uncover interdisciplinary relationships—for example, between exercise immunology and chemotherapy adherence—that may otherwise go unnoticed in conventional reviews. This method also reveals understudied topics, such as the function of exercise in metastatic breast cancer, while highlighting the prevalence of specific study themes (such as immune modulation and fatigue reduction). This study maintains attention on the specific needs of breast cancer populations while ensuring comparability with broader scientific trends by duplicating and improving approaches from well‐established bibliometric works [29, 30].

Additionally, the 2020–2024 timeframe reflects postpandemic changes in research objectives, including the emergence of fitness programs based on telemedicine and a renewed focus on mental health outcomes. According to international bibliometric evaluations [25, 26], these times of fast innovation necessitate regular monitoring in order to differentiate between fleeting trends and significant breakthroughs. The methodology′s usefulness for policymakers and clinicians looking for evidence‐based guidance is strengthened by its capacity to contextualize findings within wider scientific ecosystems, such as connecting studies on breast cancer to more general advancements in oncology rehabilitation [13]. By following these guidelines, this study ensures continuity in tracking the development of exercise oncology by mapping the current status of the field and creating a reproducible framework for upcoming updates.

## 4. Operational Definitions of Key Bibliometric Terms

### 4.1. Performance Analysis

Documents analysis: quantitative assessment of publication output (e.g., volume and growth trends) to evaluate research productivity.

Source analysis: examination of journals/conferences to identify dominant outlets in the field.

Author analysis: metrics (e.g., publication count and citations) to measure individual researcher contributions.

Organization/country analysis: geographic or institutional distribution of research activity.

#### 4.1.1. Cocitation Analysis

Mapping relationships between frequently cited pairs of publications; two documents are considered “cocited” if they appear together in the reference lists of other papers, suggesting thematic linkages.

#### 4.1.2. Co‐Occurrence Analysis

Identification of patterns where terms (e.g., keywords and author keywords) appear together in publications, revealing conceptual clusters or emerging trends.

#### 4.1.3. Coword Analysis

A subtype of co‐occurrence analysis focusing specifically on keyword networks to visualize thematic evolution and knowledge structure.

#### 4.1.4. Search Strings

In the study “Mapping the Research Landscape of Exercise and Breast Cancer: A Bibliometric Analysis From 2020 to 2024,” research trends in the relationship between physical activity and breast cancer were investigated using a systematic bibliometric approach. The first search approach focused on article titles that combined the terms “physical activity” or “exercise” with “breast cancer,” yielding 1632 papers in total. Four hundred eighty‐five pertinent publications made up the final dataset after the search was narrowed down using particular inclusion criteria.

A strict set of inclusion criteria was used to guarantee the bibliometric analysis′s validity and consistency, as shown in Table 1. The Web of Science (WoS) Core Collection was the sole source of the data, which covered the years 2020–2024. The keywords used were “physical activity” OR “exercise” AND “breast cancer,” and the search was restricted to the title field (TI). The study also used quick filters, such as language restriction to English, document type restriction to articles and review articles, and open access availability.

**Table 1:** Inclusion criteria for bibliometric analysis.

WoS database	All
Time period	2020–2024
Search field	TI
Search keywords	“physical activity” OR “exercise” AND “breast cancer”
Quick filters	Open access
Document type	Article or review article
Language	English
Web of Science index	Science Citation Index Expanded OR Social Sciences Citation Index

Additionally, the Social Sciences Citation Index (SSCI) and the Science Citation Index Expanded (SCIE) were chosen as the WoS indexes to improve the research′s academic trustworthiness. A strong basis for mapping important research trends, author partnerships, and theme hotspots in the field of exercise and breast cancer is provided by these databases, which guarantee the inclusion of high‐impact journals and academic papers.

#### 4.1.5. Findings

**Figure 1:**
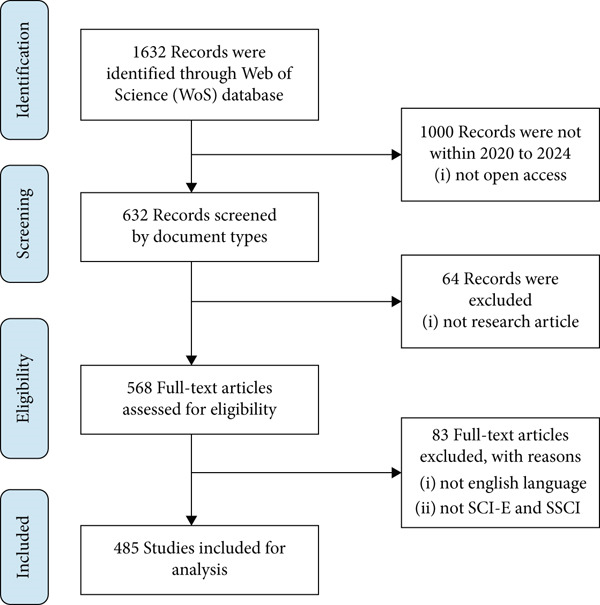
PRISMA flowchart.

The study selects bibliometric data with methodological rigor by using the PRISMA (Preferred Reporting Items for Systematic Reviews and Meta‐Analyses) methodology. The identification process started with 1632 articles that were pulled from the WoS Core Collection, as shown in Figure 1. The number of eligible publications was progressively reduced after predetermined inclusion criteria were applied, including language (English), document type (article or review), open access status, and indexation in SCIE or SSCI. A final collection of 485 studies was considered for bibliometric analysis after duplicates were eliminated and titles and abstracts were screened for relevancy. This methodical methodology guarantees that only the most relevant literature from the 2020 to 2024 timeframe was examined, improving the research′s transparency, reproducibility, and dependability.

Duplicate records were systematically identified and removed using a two‐step process: First, automated deduplication was performed within WoS based on identical titles, authors, and publication years. Second, any remaining duplicates not detected by the database (e.g., versions from different indexes) were manually checked and removed by comparing DOI, title similarity, and author lists. This rigorous approach ensured data integrity while preventing accidental exclusion of similar but distinct publications.

**Figure 2:**
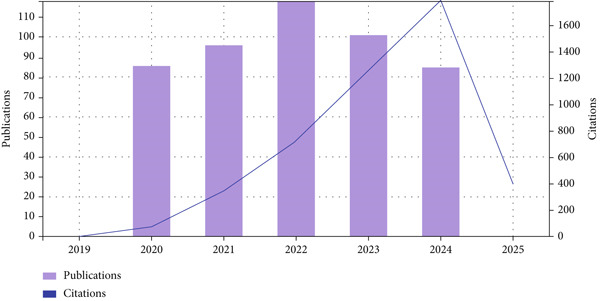
Quantity of publications and citations between 2020 and 2024.

This study′s bibliometric trend analysis provides a thorough summary of the citation dynamics and scholarly output associated with exercise and breast cancer research from 2020 to 2024. The yearly publishing volume varied slightly, reaching a peak of 118 articles in 2022 and then slightly declining in the years that followed, as shown in Figure 2. The citation count showed a consistent and noteworthy increasing tendency, demonstrating growing scholarly interest and effect within the area, despite the fluctuation in publication numbers.

In particular, the number of citations increased from 71 in 2020 to an astounding 1784 in 2024, demonstrating the maturation of research influence over time and an exponential rise in academic acknowledgment. With 1247 citations, 2023 also showed significant visibility, indicating that research from previous years kept becoming more and more relevant. Additionally, the dataset contained 485 publications with a total of 4569 citations (3928 if self‐citations are excluded), resulting in an average of 9.42 citations per item, which is a sign of the general caliber and scholarly contribution of the research.

The H‐index of 29 in terms of impact indicators supports the legitimacy and longevity of significant research in this field. The discrepancy between self‐citation–adjusted citing articles (2732) and citing articles (2973) indicates a robust citation ecosystem in which external recognition predominates. When taken as a whole, these measures show that studies relating exercise to breast cancer have developed both quantitatively and in terms of academic reach and importance, making it an essential subfield in oncology and public health research.

### 4.2. Performance Analysis

#### 4.2.1. Document Analysis

Ten papers in all reached the 60 citation inclusion requirement (Table 2), indicating their significant scholarly influence in the field of exercise and breast cancer research between 2020 and 2024. The study by Cannioto et al. [31], which looked at the connection between survival outcomes in high‐risk breast cancer patients and physical activity prior to, during, and following chemotherapy, is the most frequently mentioned. Because of its scientific integrity and therapeutic significance, this study has established itself as a foundation in the literature. Even though it is the only study in the collection with more than 100 citations, the other papers offer just as important information. For example, Hasenoehrl et al. [6] provide an updated meta‐analysis on resistance training and lymphedema, while Gomes‐Santos et al. [5] clarify how exercise alters immunological pathways to improve tumor control. When taken as a whole, these publications highlight a move toward excellent randomized trials and mechanistic research, which reflects an increasing focus on the evidence‐based incorporation of exercise into the treatment of breast cancer.

**Table 2:** Top 10 documents.

**Rank**	**Authors**	**Title**	**Citations**
1	Cannioto et al. [31]	Physical Activity Before, During, and After Chemotherapy for High‐Risk Breast Cancer: Relationships With Survival	116
2	Gomes‐Santos et al. [5]	Exercise Training Improves Tumor Control by Increasing CD8+ T‐Cell Infiltration via CXCR3 Signaling and Sensitizes Breast Cancer to Immune Checkpoint Blockade	83
3	Hasenoehrl et al. [6]	Resistance Exercise and Breast Cancer‐Related Lymphedema‐A Systematic Review Update and Meta‐Analysis	67
4	Falzone et al. [11]	Identification of Modulated MicroRNAs Associated With Breast Cancer, Diet, and Physical Activity	56
5	Khodabakhshi et al. [23]	Does a Ketogenic Diet Have Beneficial Effects on Quality of Life, Physical Activity or Biomarkers in Patients With Breast Cancer: A Randomized Controlled	56
6	An et al. [10]	Effects of Exercise Dose and Type During Breast Cancer Chemotherapy on Longer‐Term Patient‐Reported Outcomes and Health‐Related Fitness: A Randomized	49
7	Hiensch et al. [4]	Inflammation Mediates Exercise Effects on Fatigue in Patients With Breast Cancer	49
8	J. Lee and M. Lee [16]	Effects of Exercise Interventions on Breast Cancer Patients During Adjuvant Therapy: A Systematic Review and Meta‐analysis of Randomized Controlled Trials	47
9	Roberts et al. (2020)	Exercise Therapies for Preventing or Treating Aromatase Inhibitor‐Induced Musculoskeletal Symptoms in Early Breast Cancer	47
10	Koevoets et al. [14]	Effect of Physical Exercise on Cognitive Function After Chemotherapy in Patients With Breast Cancer: A Randomized Controlled Trial (PAM Study)	46

#### 4.2.2. Source Analysis

**Figure 3:**
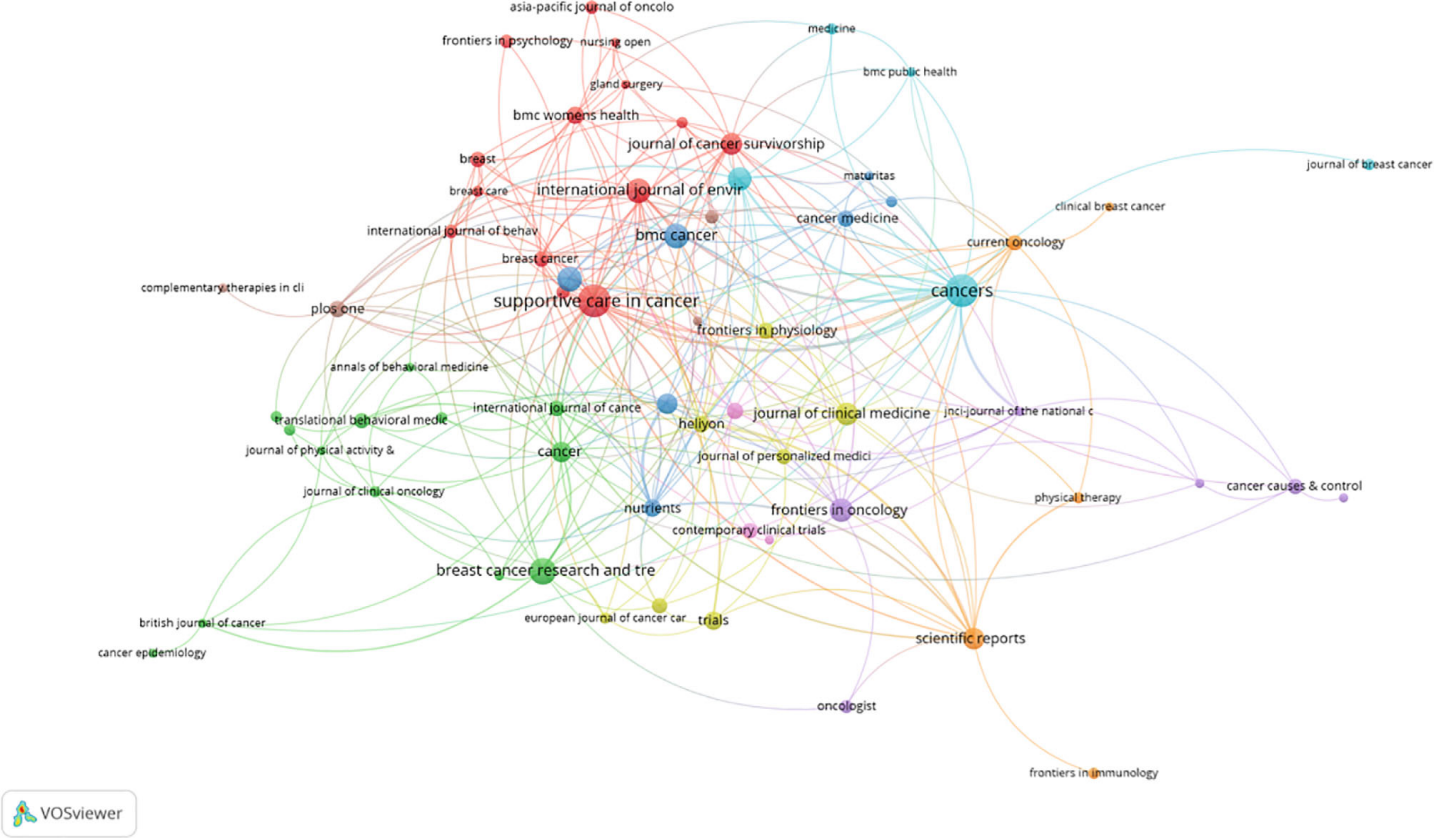
Source analysis (VOSviewer visualization).

Using a citation threshold of 64, the source analysis identifies important publications that have contributed to the spread of significant research in the field of oncology and physical exercise (Figure 3). With 24 published papers, *Supportive Care in Cancer* is the most prolific, demonstrating its dedication to disseminating research on integrative and rehabilitative oncology. Comparably, *Cancers* has 23 publications and a large number of citations, indicating that it plays a part in connecting lifestyle modifications and molecular cancer research. A significant multidisciplinary emphasis in the field is indicated by the existence of journals like *Frontiers in Oncology*, *BMC Cancer*, and *Breast Cancer Research and Treatment*. These resources encourage interdisciplinary communication between public health researchers, exercise physiologists, and oncologists in addition to offering rigorous peer‐review platforms. Exercise in breast cancer research is no longer considered a fringe topic but is instead becoming more and more integrated into mainstream oncology scholarship, as seen by their editorial reach and citation influence.

#### 4.2.3. Author Analysis

**Figure 4:**
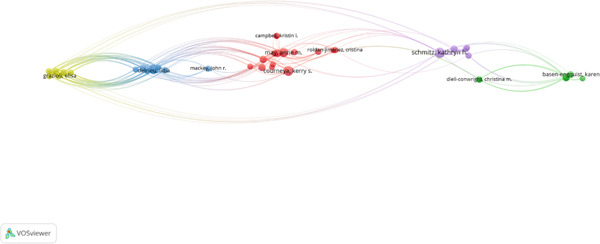
Author analysis (VOSviewer visualization).

Ten prominent authors who have made important contributions to the study on the relationship between exercise and breast cancer were found by the analysis using a threshold of 55 citations (Figure 4). With 15 papers and 137 citations, Kathryn H. Schmitz is notable for being a key player in the evolution of exercise oncology. Strong scholarly influence is also shown by Kerry S. Courneya (14 publications and 201 citations), especially through extensive clinical studies. Authors like Grazioli (177) and Dimauro (166) have a high overall link strength, which demonstrates their ability to collaborate across institutions and global networks. A notable balance between knowledge synthesis and primary data generation is demonstrated by the several eminent authors who have contributed to both empirical and review‐based literature. A mature and well‐connected academic community is reflected in this authorial landscape, where knowledge on subjects including survivorship outcomes, mechanistic insights, and the effectiveness of interventions converges.

#### 4.2.4. Organization Analysis

**Figure 5:**
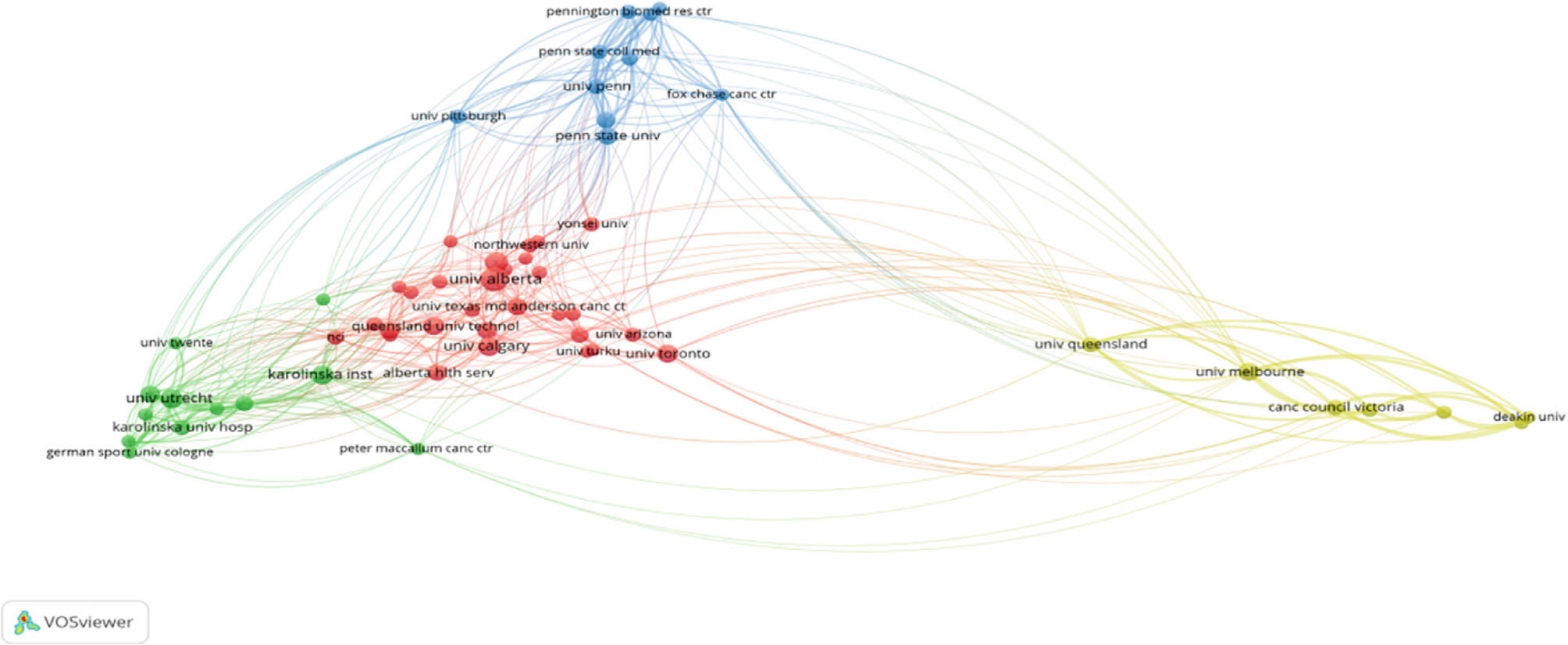
Organization analysis (VOSviewer visualization).

To find organizations that have made major contributions to the field, a citation criterion of 58 was established (Figure 5). With 21 publications and 260 citations, the University of Alberta is in the lead, demonstrating Canada′s dominant position in exercise–oncology research. Following closely behind are Karolinska Institutet (14 papers and 211 citations) and Harvard Medical School (15 documents and 229 citations), all of which have made significant investments in clinical and translational research. With total link strengths of 114 and 103, respectively, the University of Melbourne and Utrecht University stand out as having important roles in promoting global cooperation. The global dispersion of research efforts is demonstrated by the participation of institutions from North America, Europe, and Oceania. Additionally, the information suggests that the university is concentrating on multidisciplinary frameworks that integrate public health, physical therapy, and oncology.

#### 4.2.5. Country Analysis

**Figure 6:**
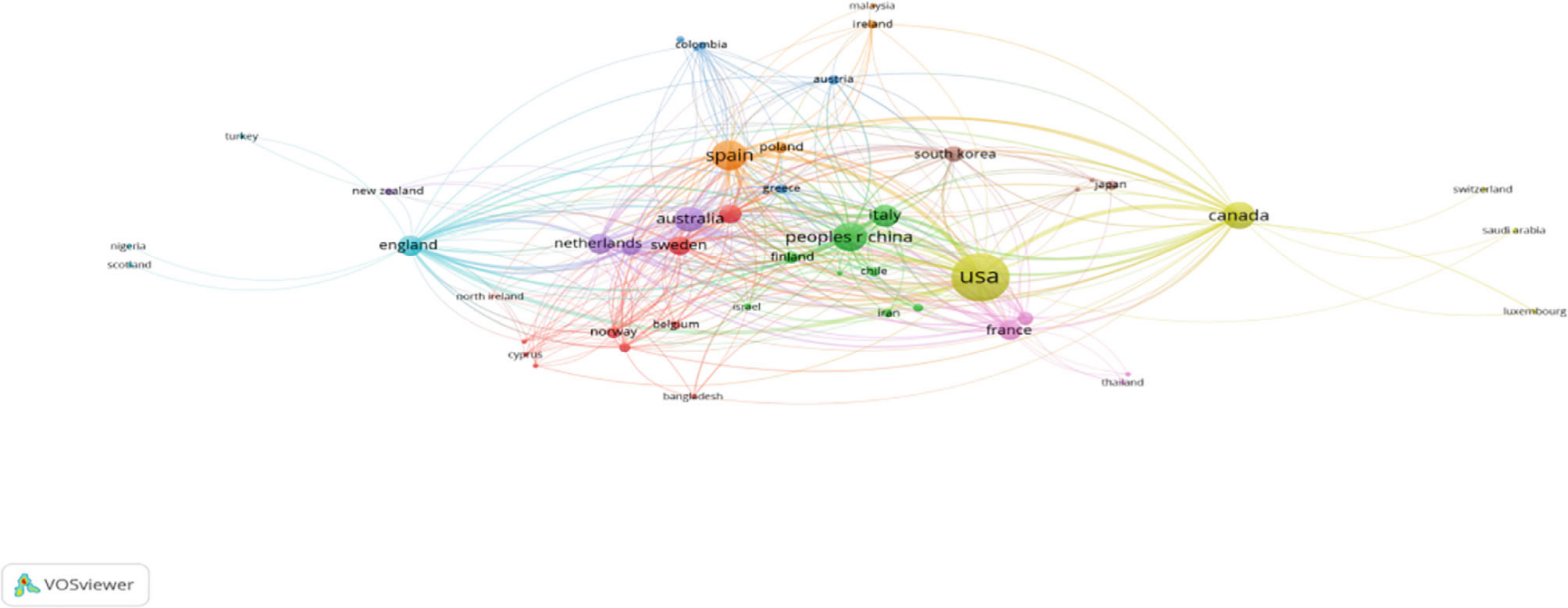
Country analysis (VOSviewer visualization).

Ten nations stood out as significant contributors to this field with a citation criterion of 49 (Figure 6). The United States leads in both research output and impact, as seen by its 162 publications and 1733 citations. The fast‐growing scholarly activity in China (60 documents and 419 citations) and Spain (61 documents and 562 citations) is bolstered by robust collaborative networks. With strong national programs promoting integrative cancer treatment, Canada (52 publications and 733 citations) and Australia (45 publications and 463 citations) also rank highly. Significant international cooperation is evident from total link strength data, particularly between European nations like the Netherlands (226) and England (207). These trends show a geographically heterogeneous and globally integrated research environment, with high‐impact countries advancing exercise‐related breast cancer research by fusing academic innovation with clinical infrastructure.

#### 4.2.6. Co‐citation Analysis

Cocitation analysis, which shows how frequently two works are referenced together, is a crucial bibliometric method for determining the intellectual underpinnings of a field of study. The 10 most cocited publications in this analysis were determined by applying a threshold of 54 citations. With 139 citations and a total link strength of 527, Campbell et al. [7] is in first place, as seen in Table 3, highlighting its critical role in developing consensus‐based exercise recommendations for cancer survivors. This article is frequently cited as a fundamental reference in both empirical and conceptual research, and it represents an international, multidisciplinary attempt to standardize physical activity recommendations. With 82 citations and a link strength of 357, Schmitz et al. [32] come next, demonstrating the guidelines′ continued applicability. When taken as a whole, these two works formalize exercise oncology and are often mentioned alongside new empirical research, demonstrating their importance in influencing later investigations.

**Table 3:** Co‐citations (Top 10 articles).

**Rank**	**Authors**	**Title**	**Citations**	**Total link strength**
1	Campbell et al. [7]	Exercise Guidelines for Cancer Survivors: Consensus Statement From International Multidisciplinary Roundtable	139	527
2	Schmitz et al. [32]	American College of Sports Medicine Roundtable on Exercise Guidelines for Cancer Survivors	82	357
3	Aaronson et al. [33]	The European Organization for Research and Treatment of Cancer QLQ‐C30: A Quality‐of‐Life Instrument for Use in International Clinical Trials in Oncology	59	194
4	Bray et al. (2020)	Erratum: Global Cancer Statistics 2018: GLOBOCAN Estimates of Incidence and Mortality Worldwide for 36 Cancers in 185 Countries	49	177
5	Courneya et al. [2]	Effects of Aerobic and Resistance Exercise in Breast Cancer Patients Receiving Adjuvant Chemotherapy: A Multicenter Randomized Controlled Trial	46	158
6	Lahart et al. [20]	Sports Analytics and the Big‐Data Era	44	232
7	Furmaniak et al. [34]	Exercise for Women Receiving Adjuvant Therapy for Breast Cancer	42	197
8	Dieli‐Conwright et al. [9]	Aerobic and Resistance Exercise Improves Physical Fitness, Bone Health, and Quality of Life in Overweight and Obese Breast Cancer Survivors: A Randomized Controlled Trial	41	213
9	Schmitz et al. [8]	Exercise Is Medicine in Oncology: Engaging Clinicians to Help Patients Move Through Cancer	38	204
10	Holmes et al. [3]	Physical Activity and Survival After Breast Cancer Diagnosis	38	159

Aaronson et al. [33], who introduced the EORTC QLQ‐C30 quality‐of‐life instrument, are the third most cocited work. Despite being older, its 194 link strengths and 59 citations highlight its ongoing relevance in clinical trials and intervention evaluations involving populations with breast cancer. Notable for providing a worldwide epidemiological perspective using GLOBOCAN data, Bray et al. [1] are frequently referenced in research addressing the incidence of breast cancer and the justification for lifestyle‐based therapies. High‐quality RCTs that offer compelling evidence for the physiological and psychological advantages of aerobic and resistance training may be found in articles like Courneya et al. [2] and Dieli‐Conwright et al. [9]. Their ranking among the Top 10 serves as additional evidence of the scientific basis for exercise recommendations. Crucially, their overall link strengths (158 and 213, respectively) confirm their methodological and conceptual compatibility by indicating that they frequently coappear with guideline literature.

By addressing systematic reviews, survivorship outcomes, and clinical advocacy for exercise integration, the following articles—such as Lahart et al. [20], Furmaniak et al. [34], Schmitz et al. [8], and Holmes et al. [3]—expand the theoretical and clinical framework. Holmes et al. offer early observational data that link physical exercise to survival, while Lahart et al.′s meta‐analysis and Furmaniak′s Cochrane review combine evidence on mortality and recurrence risk. A closely knit citation network is indicated by the sum of the link strengths between these publications, confirming their combined impact on the field′s knowledge structure. A coherent and integrated body of knowledge in exercise and breast cancer research is reflected in the cocitation map, which shows a mature and evidence‐driven academic domain where foundational guidelines, validated instruments, and high‐level trials are frequently cited together.

#### 4.2.7. Co‐citation Analysis by Clusters

**Figure 7:**
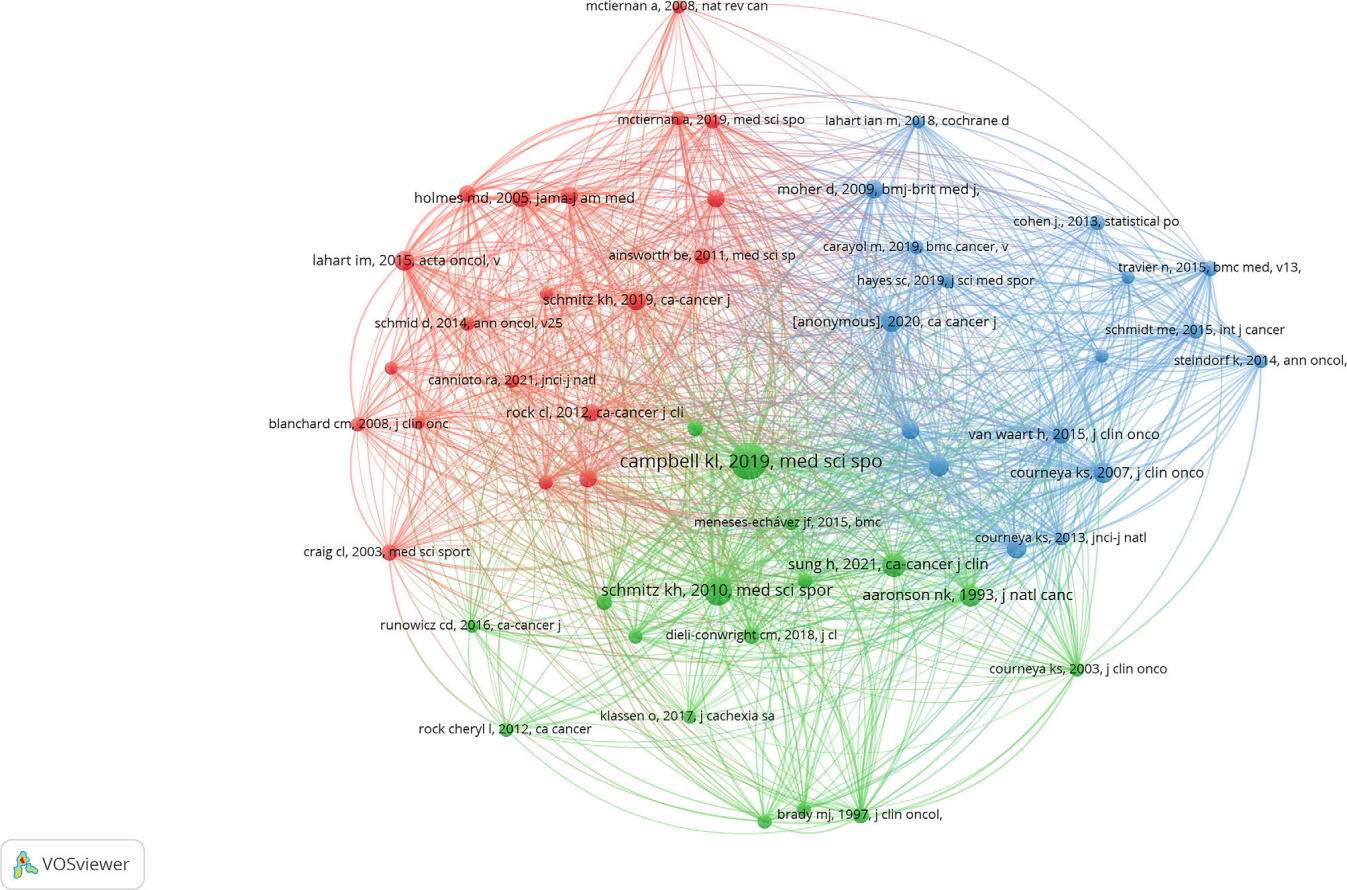
Co‐citation analysis (VOSviewer visualization).

A thorough understanding of the intellectual framework in the study of exercise and breast cancer can be obtained by cocitation analysis. This approach identifies clear clusters of regularly cocited references that identify key theme regions, as seen in Figure 7. Based on cocitation frequency, Table 4 shows three major clusters: blue (Cluster 3), green (Cluster 2), and red (Cluster 1). To guarantee that each cluster represents significant contributions to the field, a criterion was set to include only highly cited references. These clusters, which connect epidemiology, cancer, exercise science, and survivorship research, show a high level of interdisciplinary collaboration. Together, they demonstrate how exercise is becoming seen as an essential part of cancer treatment and survivorship planning, rather than just a supportive therapy. This analysis demonstrates the field′s conceptual development and emphasizes how clinical interventions and public health viewpoints have converged in the treatment of breast cancer.

**Table 4:** Co‐citation cluster.

**Cluster no. and color**	**Cluster labels**	**No. of articles**	**Representative publications**
Cluster 1 (red)	Survivorship and physical activity guidelines	20	Lahart et al. [20]; Schmitz et al.[8]; Holmes et al.[3]; Patel et al. [15]; Spei et al. [19]; Rock et al. [35]; Irwin et al. (2003); Craig et al. [36]; Ainsworth et al. [37]; Ibrahim and Al‐Homaidh (2011)
Cluster 2 (green)	Quality of life and clinical guidelines	17	Campbell et al. [7]; Schmitz et al. [32]; Sung et al. [22]; Aaronson et al. [33]; Brady et al. [38]; Dieli‐Conwright et al. [9]; Speck et al. (2010); Ml. (2006) Rogers et al. [39]
Cluster 3 (blue)	Intervention efficacy and meta‐analysis	17	Bray et al. (2020); Courneya et al. [2]; Furmaniak et al. [34]; Dieli‐Conwright et al. [9]; Moher et al. [18]; Van Waart et al. [24]; Juvet et al. [40]; Schmidt et al. (2015); Travier et al. (2015)

The largest group, Cluster 1, consists of 20 important publications and is mostly concerned with physical exercise recommendations and long‐term results for breast cancer survivors. Lahart et al. [20], Holmes et al. [3], and Schmitz et al. [8] are representative studies that examine the relationship between regular physical exercise and increased survival and decreased recurrence. Reputable standards for including exercise and nutrition into survivorship treatment are further established by publications such as Patel et al. [15] and Rock et al. [35]. The empirical base of this cluster is shown by the inclusion of fundamental methodological tools like the Compendium of Physical Activities [37] and the International Physical Activity Questionnaire [36]. This theme focus exemplifies a paradigm change in oncology, where exercise is operationalized as a quantifiable, recommended intervention. These studies′ foundational importance in forming survivorship procedures is further supported by the threshold for inclusion in this cluster, which probably reflects both methodological impact and high citation volume.

Cluster 2, which has 17 papers, focuses on standardizing exercise programs, clinical guidelines, and quality‐of‐life evaluations. In exercise oncology, key references like Campbell et al. [7] and Schmitz et al. [32] reflect attempts to reach a consensus. Measurement instruments such as the FACT‐B [38] and the EORTC QLQ‐C30 [33] demonstrate a considerable focus on psychometric evaluation in intervention studies. Epidemiological and clinical viewpoints on the effects of structured exercise programs on metabolic and psychosocial outcomes in breast cancer survivors are provided by Sung et al. [22] and Dieli‐Conwright et al. [9]. High cocitation frequency established the threshold in this case, highlighting the need for patient‐centered care and intervention design relevance. The cluster points to a developing topic that reflects the increasing need for comprehensive cancer management strategies by giving equal weight to subjective and physiological outcomes.

The 17 publications in Cluster 3 are devoted to systematic reviews, methodological consistency, and the effectiveness of interventions in exercise–oncology trials. Influential works that examine the effects of aerobic and resistance exercise both before and after therapy include Bray et al. [1], Courneya et al. [2], and Furmaniak et al. [34]. Notably, the PRISMA framework is established by Moher et al. [18], highlighting the methodological rigor in meta‐analyses that comprise this cluster′s core. The inclusion of research such as van Waart et al. [24] and Juvet et al. [40] shows how the timing and intensity of exercise impact fatigue reduction and treatment adherence. This group′s threshold highlights the methodological authority and translational value of articles that are regularly cited in intervention trials and reviews. This cluster summarizes the body of research supporting the clinical use of exercise in controlling side effects and improving therapeutic results when incorporated into treatment plans.

#### 4.2.8. Co‐Occurrence Analysis

By analyzing the frequency with which keywords occur together in the literature, the co‐occurrence analysis—a fundamental component of bibliometric mapping—identifies the thematic landscape of research. A targeted collection of high‐frequency phrases was obtained by setting a threshold of 61. A total of 1726 distinct keywords were found in the dataset, suggesting a wide range of themes. With a total link strength of 7799, these keywords created 1415 linkages that represented co‐occurrence relationships. The significant connections between important research issues in the area of exercise and breast cancer are highlighted by this high link strength. Studies in this field frequently conceive exercise within the larger clinical and psychosocial context of breast cancer, as shown by the core nodes of “breast cancer” and “exercise” in Table 5. The network serves as an example of a developing field that is distinguished by interdisciplinary integration that is cohesive and terminology that is becoming more standardized.

**Table 5:** The 15 most frequent keywords in the co‐occurrence analysis.

**Rank**	**Keyword**	**Occurrences**	**Total link strength**
1	breast cancer	222	1172
2	exercise	198	1088
3	quality‐of‐life	148	905
4	physical activity	144	778
5	survivors	133	824
6	women	118	712
7	physical‐activity	104	601
8	fatigue	95	606
9	health	75	462
10	risk	68	372
11	quality of life	64	402
12	Meta analysis	64	398
13	chemotherapy	61	347
14	guidelines	51	302
15	aerobic exercise	45	259

Among the most commonly used terms, “exercise” (198; 1088) and “breast cancer” (222 occurrences; total link strength: 1172) obviously dominate the thematic structure. Their close relationship emphasizes how important exercise is as a treatment and prevention strategy in the literature on breast cancer. Both “physical activity” (144; 778) and “quality‐of‐life” (148; 905) are highly ranked, indicating a definite focus on behavioral therapies and patient‐centered outcomes. Interestingly, the phrase “women” (118; 712) emphasizes the gendered focus inherent in breast cancer research, while the term “survivors” (133; 824) represents the growing move toward posttreatment care. The fact that “physical activity” and “physical‐activity” appear as distinct phrases may indicate inconsistent keyword standards, but taken together, they strengthen the focus on movement‐based interventions. These key concepts show how lived experiences, behavioral frameworks, and survivorship narratives influence the research environment in addition to clinical outcomes.

A closer examination of less common but important keywords, such as “fatigue” (95; 606), “risk” (68; 372), and “chemotherapy” (61; 347), suggests a focus on risk assessment and side effect mitigation. A shift from exploratory research to guideline‐driven research is shown by the terms “meta‐analysis” (64; 398) and “guidelines” (51; 302), which emphasize an increasing emphasis on the synthesis of data and the formalization of clinical recommendations. Notably, “aerobic exercise” (45; 259) suggests that future studies may increasingly differentiate across exercise modalities, indicating greater interest in intervention specificity. The persistence of both physiological and methodological words points to a research setting that embraces more general public health objectives while aiming for clinical precision. All things considered, the co‐occurrence patterns suggest that the conversation on exercise therapies in the treatment of breast cancer is becoming more organized and evidence‐based.

#### 4.2.9. Coword Analysis

**Figure 8:**
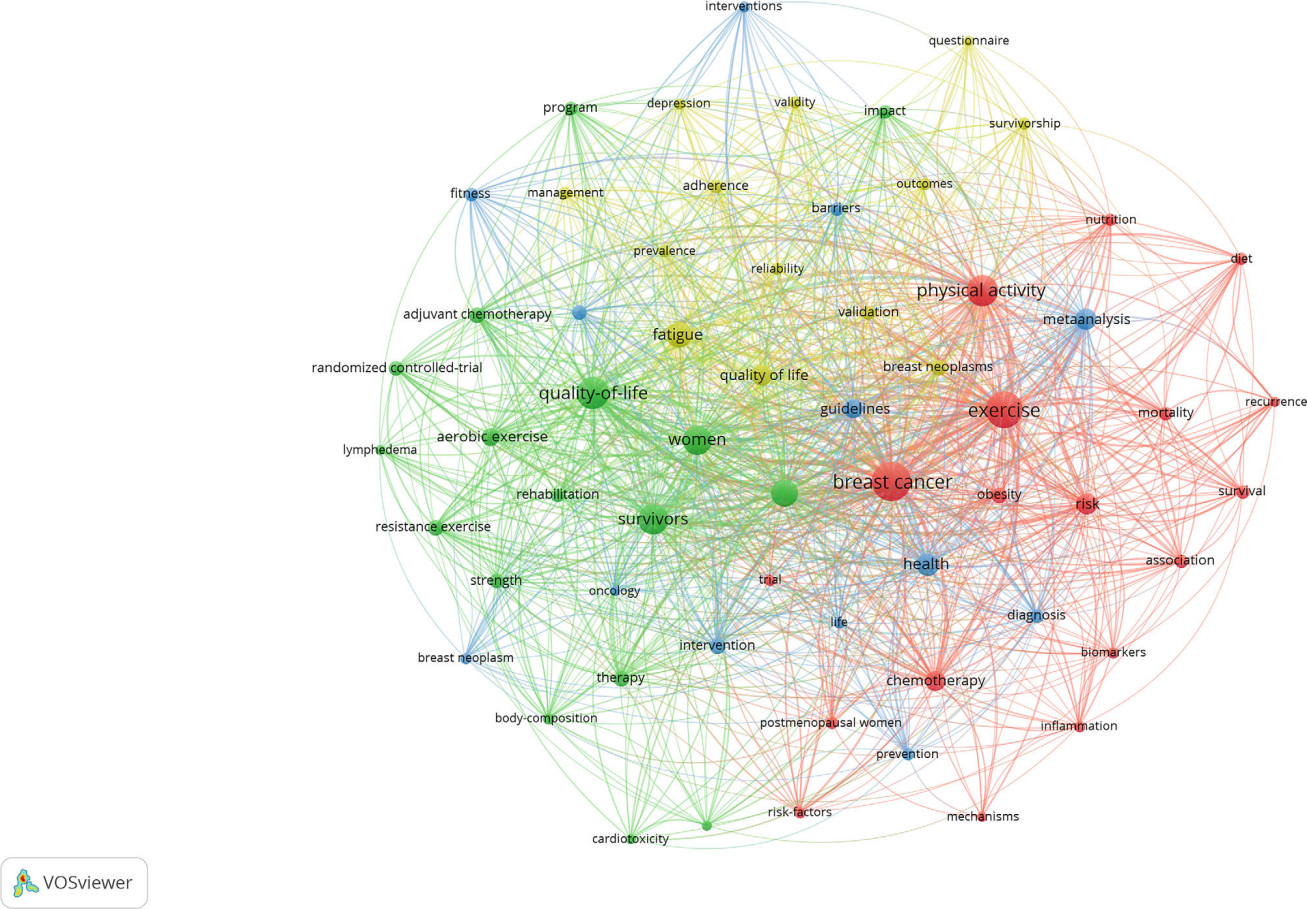
Co‐word analysis of exercise and breast cancer (VOSviewer visualization).

Four unique thematic clusters formed from keyword co‐occurrence in the field of exercise and breast cancer from 2020 to 2024 are revealed by the coword analysis, which is summarized in Table 6 and illustrated in Figure 8. Based on their colinkage patterns, 61 high‐frequency keywords were categorized into red, green, blue, and yellow clusters using the VOSviewer method. This method reveals underlying research themes and conceptual frameworks that mirror the changing conversation at the nexus of exercise science and oncology. The network shows a multifaceted study focus with keywords like “breast cancer,” “exercise,” “quality‐of‐life,” and “meta analysis” showing up across clusters. A balanced focus on clinical outcomes, intervention techniques, and psychological aspects is also evident in the keyword distribution. In addition to mapping key ideas, the clusters provide information on interdisciplinary priorities such as methodological development, treatment adherence, and survivability.

**Table 6:** Co‐word analysis on the relationship between exercise and breast cancer.

**Cluster no. and color**	**Cluster label**	**Number of keywords**	**Representative keywords**
1 (red)	Clinical risk and metabolic outcomes	18	“breast cancer,” “exercise,” “physical activity,” “risk,” “chemotherapy,” “obesity,” “association,” “mortality,” “nutrition”
2 (green)	Exercise modalities and survivorship	17	“quality‐of‐life,” “survivors,” “women,” “physical‐activity,” “aerobic exercise,” “therapy,” “resistance exercise,” “adjuvant chemotherapy,” “strength”
3 (blue)	Evidence synthesis and implementation	13	“health,” “meta analysis,” “guidelines,” “intervention,” “diagnosis,” “breast cancer survivors,” “barriers”
4 (yellow)	Psychosocial impact and measurement validity	13	“fatigue,” “quality of life,” “breast neoplasms,” “adherence,” “validation,” “prevalence,” “validity”

With 18 terms, Cluster 1 focuses on the relationship between exercise, metabolic health, and breast cancer. The use of physical activity as a preventative and therapeutic strategy to lower cancer‐related risk factors is strongly emphasized in key terms like “breast cancer,” “exercise,” “physical activity,” and “risk.” The use of words like “obesity,” “nutrition,” “mortality,” and “chemotherapy” indicates that breast cancer research has metabolic and treatment‐related components. This cluster indicates an increasing interest in learning how lifestyle factors that can be changed affect the course of disease and the prognosis of patients. It also emphasizes how exercise may be included in treatment plans and primary prevention approaches. The word “association” refers to the widespread use of observational study methods to investigate connections between clinical endpoints and behavioral factors. The significance of lifestyle interventions in lowering disease burden and enhancing survival outcomes is a fundamental problem in clinical oncology and public health that is collectively captured by this cluster.

The 17 keywords in Cluster 2, “exercise modalities and survivorship,” are centered on the lived experiences of breast cancer survivors and the kinds of physical activity therapies that are used. Phrases like “quality‐of‐life,” “survivors,” “women,” and “therapy” draw attention to a patient‐centered and gendered study focus. Meanwhile, the terms “aerobic exercise,” “resistance exercise,” and “strength” highlight the importance of adjusting exercise regimens to suit each patient’s requirements and stage of therapy. “Adjuvant chemotherapy” reinforces the importance of structured physical exercise in posttreatment care by tying therapeutic treatments to recovery techniques. This cluster represents an emerging line of research that connects cancer rehabilitation and exercise physiology. The cluster exemplifies the importance of integrative oncology treatments that go beyond tumor care to long‐term wellness and functionality by placing a high priority on survivorship and quality‐of‐life outcomes.

Cluster 3, which consists of 13 terms, is titled “evidence synthesis and implementation.” The terms “health,” “meta analysis,” “guidelines,” and “intervention” all point to a scientific endeavor to convert amassed data into useful clinical procedures. Words like “diagnosis,” “breast cancer survivors,” and “barriers” allude to practical implementation issues like access and exercise program adherence. The transition from experimental design to standardization and clinical integration is exemplified by this cluster. The use of “guidelines” is a reflection of the increasing weight that policy texts and expert consensus are giving to initiatives. Through systematic reviews and meta‐analyses, it also demonstrates a dedication to methodological rigor, solidifying this cluster as the foundation for evidence‐based decision‐making in exercise oncology.

There are 13 keywords in Cluster 4, “psychosocial impact and measurement validity,” which is concerned with the assessment of behavioral and psychological aspects of breast cancer treatment. Concerns about symptom load and patient participation are indicated by keywords like “fatigue,” “adherence,” and “quality of life.” As researchers work to guarantee the reliability of outcome measures used in clinical and behavioral investigations, the terms “validation,” “validity,” and “prevalence” imply a methodological foundation. The addition of “breast neoplasms” strengthens the practical significance of these ideas by connecting them to diagnostic frameworks. This cluster demonstrates how clinical practice and measurement science combine to improve instruments for evaluating treatment impact, adherence, and well‐being. It highlights the need for both successful interventions and proven techniques to evaluate their psychological efficacy in order to make progress in this area.

## 5. Discussion

The research landscape on exercise and breast cancer from 2020 to 2024 was methodically mapped out by this bibliometric analysis, which identified a number of important patterns. Citations in the subject have increased exponentially, and scholarly impact has been driven by foundational publications like Cannioto et al. [31] and Gomes‐Santos et al. [5]. Three topic clusters serve as the foundation for the conceptual structure: quality‐of‐life and therapeutic interventions, intervention efficacy, and survivorship and physical activity guidelines. Influential writers like Kathryn H. Schmitz and high‐impact journals like *Supportive Care in Cancer* highlight how exercise science is incorporated into oncology. Geographical differences were apparent, with Western countries producing the majority of research; however, new contributions from Asia and Europe point to a slow change. Coword analysis revealed changing objectives that reflected a shift from exploratory research to guideline‐driven therapies, such as metabolic health, exercise modalities, and evidence synthesis.

This study′s bibliometric review of the literature on exercise and breast cancer provides researchers, doctors, and healthcare policymakers with important new information. The results highlight a number of crucial areas that need focus, including strengthening interdisciplinary teamwork, advocating for evidence‐based exercise regimens, and including physical activity into all‐encompassing cancer treatment plans. Optimizing physical and psychological well‐being requires putting patient‐centered outcomes first, encouraging long‐term survivorship, and customizing exercise regimens to meet specific clinical requirements. Furthermore, the focus on individualized exercise regimens to suit various treatment phases and health characteristics highlights the significance of comprehensive and flexible approaches in oncological rehabilitation.

### 5.1. Theoretical Implications

By clarifying the connections between physical activity and tumor biology and systemic physiology, this bibliometric analysis contributes to theoretical frameworks in exercise oncology. A paradigm change from considering exercise as supportive care only to acknowledging its significance as a modulator of cancer progression is revealed by the cocitation clusters. For example, exercise‐induced microRNAs that control tumor microenvironments were discovered by Falzone et al. [11], pointing to molecular reasons behind the survival improvements seen in epidemiology studies [3]. Similar to this, immunological regulation via increased CD8+ T‐cell infiltration [5] bridges the gap between mechanistic theories and empirical facts by offering a biological basis for exercise′s therapeutic potential. In order to understand exercise′s complex impacts on breast cancer outcomes, theoretical models that incorporate its pleiotropic effects—which include metabolic reprogramming, inflammation reduction, and epigenetic regulation—are required.

In order to handle dose–response heterogeneity and customized intervention design, the analysis emphasizes the necessity for improved theoretical frameworks. Although the benefits of exercise for survival were demonstrated by population‐level studies [20], less is known about the diversity of individual responses, which can be altered by tumor subtypes, treatment modalities, and genetic variables. Ecological models that incorporate social, psychological, and physiological factors, as Spei et al. [19], provide a basis for predicting the best exercise parameters but are not very detailed. By taking into consideration adherence obstacles and cognitive–behavioral mediators, biobehavioral theories such as Rogers et al.′s [39] social cognitive framework could improve accuracy. In order to create adaptive models that direct individualized exercise prescriptions across a range of clinical scenarios, future theoretical work must balance these multilayer aspects.

Lastly, the bibliometric mapping identifies areas where cross‐disciplinary theoretical integration may be possible. Coword clusters (Cluster 1) that associate exercise with metabolic health and survivorship demonstrate a comprehensive strategy that combines public health and oncology viewpoints. Mechanistic studies (Cluster 3) emphasize the need to integrate molecular insights with patient‐centered outcomes, which is consistent with translational research paradigms. A theoretical drive toward standardization in exercise cancer research is further indicated by the prevalence of guidelines such as PRISMA [18] in methodological clusters. In order to promote a cohesive theoretical framework that views exercise as a population health strategy and a precision therapeutic tool in the therapy of breast cancer, this study encourages communication between epidemiologists, molecular biologists, and behavioral scientists.

### 5.2. Practical Implications

The bibliometric analysis emphasizes how urgently standardized exercise regimens are needed in the treatment of breast cancer. Although previous research has demonstrated the advantages of both resistance and aerobic training, there are still discrepancies in the definitions of exercise modality, duration, and intensity [7, 32]. For example, there are gaps in specialized advice due to the preponderance of survivorship‐focused research (Cluster 1) and the lack of focus on metastatic populations. In order to standardize exercise recommendations throughout therapy phases, clinicians ought to embrace evidence‐based recommendations, as those put forth by Patel et al. [15]. Thematic clusters in VOSviewer also highlight the effectiveness of resistance training in reducing lymphedema [6], calling for its incorporation into rehabilitation regimens. Standardization will guarantee that exercise becomes a regular part of oncology practice by improving repeatability and facilitating cross‐study comparisons.

One important factor in converting research into clinical practice is interdisciplinary collaboration. Journals such as *Cancers* and *Supportive Care in Cancer* are recognized by the cocitation network as links between exercise science and molecular oncology [12, 13]. For instance, in order to maximize exercise timing during immunotherapy, clinical trials must be informed by mechanistic investigations on immune regulation [5]. Similar to this, collaborations between exercise physiologists and oncologists could use customized regimens to address adherence issues, including fatigue management [4]. Institutions like the University of Alberta are highlighted in VOSviewer′s cooperation maps as centers for these kinds of programs, indicating that international knowledge‐sharing platforms may hasten implementation.

To ensure equitable exercise oncology, socioeconomic gaps must be addressed. The literature′s underrepresentation of low‐resource settings [17] reflects accessibility issues in the real world, where participation is restricted by cost and facility access [9]. According to Cormie et al. [21], community‐based initiatives show how telehealth and mobile health technologies can democratize access to exercise interventions. Funding for scalable solutions should be given top priority by policymakers, especially in areas where the incidence of breast cancer is high. Furthermore, referral rates might be raised by clinician education programs that highlight the benefits of exercise in lowering the risk of recurrence [20]. Exercise oncology can reduce systemic disparities by coordinating research efforts with public health requirements.

With ramifications for clinical practice and policy, the bibliometric analysis emphasizes the increasing focus on physical exercise in breast cancer research. Research like that conducted by Campbell et al. [7] and Schmitz et al. [8] highlights the necessity of incorporating exercise recommendations into care regimens for cancer survivors. The implementation of structured exercise programs in therapeutic settings is supported by the constant findings of increased quality of life, decreased fatigue, and improved survival rates (e.g., Cannioto et al. [31] and J. Lee and M. Lee [16]). To ensure that cancer patients receive accessible and customized rehabilitation, policymakers should promote multidisciplinary teamwork and give priority to supporting community‐based programs [21].

Lastly, maintaining patient interaction requires utilizing technologies and flexible frameworks. Opportunities to include digital tools into fitness programs are presented by the postpandemic surge in telehealth, which is represented in keyword trends [26]. For example, virtual reality platforms could mimic supervised training for patients in rural areas, and mobile apps that assess physical activity could improve adherence among survivors [24]. Furthermore, dynamic monitoring techniques are required to evaluate the long‐term benefits of exercise due to bibliometric gaps in long‐term follow‐up studies [40]. Clinicians may overcome logistical obstacles and guarantee that exercise therapies are available, customized, and in line with changing patient needs by implementing agile approaches and digital technologies.

### 5.3. Comparison With Prior Studies

The results are consistent with earlier bibliometric research, like Fresno‐Alba et al. [12], which found survivorship and exercise immunology to be the most prevalent themes. However, in contrast to previous studies that focused on more comprehensive lifestyle interventions, this study specifically underscores the growing emphasis on individualized exercise prescriptions and digital health integration. Variations in cluster identification when compared to research using CiteSpace may be explained by methodological changes, such as the use of VOSviewer for cocitation analysis. In contrast to previous assessments that gave priority to clinical results, the significance of mechanistic studies, including the role of microRNAs [11], suggests that the field is maturing toward translational science.

### 5.4. Optimizing Exercise–Oncology Integration

Exercise may improve the effectiveness of immunotherapy for breast cancer, according to new research. Exercise training enhanced CD8+ T‐cell infiltration through CXCR3 signaling, making tumors more susceptible to immune checkpoint inhibition, as Gomes‐Santos et al. [5] showed. Exercise may alter the tumor microenvironment, according to preclinical research [11]. Testing combination exercise–immunotherapy protocols should be the main focus of future trials, especially for triple‐negative breast cancer, where immunotherapy is being used more and more.

Though the ideal dosage (intensity/duration) is still unknown, current research suggests structured aerobic and resistance training [2, 10]. Although the ACSM roundtable [7] suggests 150+ min of moderate activity each week, individualized prescriptions are required, taking into account the toxicity and treatment phase. For instance, aerobic exercise lowers fatigue [4], but resistance training reduces lymphedema [6]. To increase adherence, clinicians could incorporate exercise oncology into routine therapy while utilizing digital tools (such as applications for remote monitoring).

There are still significant disparities among older, socioeconomically diverse, and metastatic groups. Despite having a greater incidence of breast cancer, older persons are underrepresented in exercise trials, and only 12% of these trials include metastatic patients [19, 22]. To get over obstacles like cost and accessibility, community‐based interventions [21] and policy‐level solutions (supported programs, for example) are crucial. To guarantee equitable benefits, these populations must be given priority in future research.

### 5.5. Limitations

This study′s shortcomings include its reliance on WoS‐indexed English publications, which may have resulted in the omission of regional contributions, despite the advantages of rigorous PRISMA‐guided data extraction and the powerful visualization provided by VOSviewer. While the 2020–2024 timeframe catches postpandemic patterns, it might miss longer term changes. In order to evaluate social impact, future studies should use other metrics (such as Altmetrics) and broaden the scope of data sources to include Scopus or gray literature. Although it ensures quality, the removal of non‐peer‐reviewed outputs may skew results in favor of well‐established institutions. The study′s limited timeline, which might not adequately capture new trends, is its main drawback. Furthermore, the requirement for uniform nomenclature is highlighted by keyword variety (e.g., “physical activity” vs. “physical‐activity”).

### 5.6. Future Avenues

To better detect and predict new trends in exercise oncology, future studies should combine conventional bibliometric approaches with cutting‐edge analytical techniques like machine learning‐based citation prediction. Qualitative content analysis should be used in conjunction with this to investigate contextual factors, such as socioeconomic and cultural barriers, that impact exercise adherence. Monitoring emerging hotspots, such as telehealth‐based therapies and biomarker‐guided exercise prescriptions, requires longitudinal observation. To encourage more inclusive global research, special attention should be paid to growing international collaboration networks with underrepresented regions. In order to preserve bibliometric objectivity and enhance qualitative analysis of implementation contexts and clinical translation factors—particularly with regard to the applicability of exercise interventions across various healthcare systems and cultural backgrounds—a mixed‐methods approach is advised. More thorough evidence for individualized exercise prescription methods may also be obtained by creating multimodal databases that combine clinical trial data, empirical evidence, and patient‐reported outcomes.

## 6. Conclusion

From 2020 to 2024, this bibliometric study outlines the changing research goals and cooperation networks in the field of exercise and breast cancer. The topic has grown significantly, as seen by rising citations and high‐impact papers on immunological regulation, exercise recommendations, and survivorship. Cocitation analysis reveals foundational guidelines [7] and mechanistic research driving innovation, while journals like *Supportive Care in Cancer* and prominent writers like Kathryn H. Schmitz highlight the integration of exercise science into oncology. Disparities still exist, though, with low‐resource environments, resistance training, and metastatic populations continuing to be underrepresented. The need for global parity in exercise cancer activities is further indicated by the dominance of high‐income nations in research production.

Though practical translation is hampered by gaps in dose–response frameworks and tailored models, theoretical advances highlight exercise as a modulator of tumor biology and patient‐reported outcomes. Future studies must balance population‐level data with precision‐based methodologies, even though ecological and biobehavioral theories offer a basis. Thematic clusters relating exercise to survivorship, metabolic health, and evidence synthesis are highlighted by coword analysis, which reflects a move away from exploratory research and toward interventions guided by guidelines. In order to integrate mechanistic findings with real‐world applications and guarantee that exercise is acknowledged as a targeted therapeutic modality as well as a public health strategy, this evolution emphasizes the significance of interdisciplinary collaboration.

This study has limitations despite its contributions, such as its reliance on English publications indexed by WoS and its limited duration (2020–2024), which may cause it to miss regional contributions or longer‐term trends. To put bibliometric results in context, future studies should use a variety of approaches and broaden their data sources (such as Scopus and gray literature). The study′s findings support exercise oncology as a vital part of all‐encompassing cancer therapy by providing practical recommendations for clinical practice, financial distribution, and international equality programs. Transdisciplinary cooperation and fair application will be essential as the subject develops in order to convert research into real patient benefits on a global scale.

In practical terms, the results support the use of organized exercise regimens in clinical care, especially for the management of fatigue, lymphedema, and toxicities associated with treatment. Randomized trials showing increased survival and quality of life highlight the importance of resistance and aerobic exercise. Future studies should explore the synergistic effects of exercise combined with emerging therapies, such as immunotherapy, to optimize multimodal treatment protocols. However, policy‐level solutions, such as financing community programs and digital health tools, are needed to address systemic impediments, such as inadequate infrastructure and clinician engagement. Further research could also investigate how exercise–immunotherapy interactions may enhance patient outcomes in diverse socioeconomic and healthcare settings. Exercise oncology may move from evidence to practice and improve comprehensive breast cancer care globally by emphasizing patient‐centered outcomes, encouraging international collaboration, and resolving implementation issues.

## Disclosure

All authors read and approved the final manuscript. All authors commented on previous versions of the manuscript.

## Conflicts of Interest

The authors declare no conflicts of interest.

## Author Contributions

All authors contributed to the study conception and design. Writing—original draft preparation: Jian Li. Writing—review and editing: BinBin Zhang. Conceptualization: BinBin Zhang. Methodology: Jian Li. Formal analysis and investigation: Jian Li. Funding acquisition: BinBin Zhang. Resources: LiYing Qiang. Supervision: MingYue Jiao. Jian Li and LiYing Qiang contributed equally to this work and share first authorship.

## Funding

This study was supported by the 2025 Shanxi Province Higher Education Institutions Teaching Reform and Innovation Project (Project Number: J20250355) and Ministry of Education Industry‐University Collaborative Talent Development Project (Project No. 230803579234630).

## Data Availability

The datasets used and/or analyzed during the current study are available from the corresponding author upon reasonable request. The data supporting this study were retrieved from the Web of Science (WoS) Core Collection using the following advanced search query: TI = (“physical activity” OR “exercise” AND “breast cancer”).
